# *Wolbachia*: endosymbiont of onchocercid nematodes and their vectors

**DOI:** 10.1186/s13071-021-04742-1

**Published:** 2021-05-07

**Authors:** Ranju Ravindran Santhakumari Manoj, Maria Stefania Latrofa, Sara Epis, Domenico Otranto

**Affiliations:** 1 Department of Veterinary Medicine, University of Bari, Valenzano, Italy; 2 Department of Biosciences and Pediatric CRC ‘Romeo Ed Enrica Invernizzi’, University of Milan, Milan, Italy; 3 Faculty of Veterinary Sciences, Bu-Ali Sina University, Hamedan, Iran

**Keywords:** *Wolbachia*, Endosymbionts, Onchocercid nematodes, Vector, Treatment, Control

## Abstract

**Background:**

*Wolbachia* is an obligate intracellular maternally transmitted, gram-negative bacterium which forms a spectrum of endosymbiotic relationships from parasitism to obligatory mutualism in a wide range of arthropods and onchocercid nematodes, respectively. In arthropods *Wolbachia* produces reproductive manipulations such as male killing, feminization, parthenogenesis and cytoplasmic incompatibility for its propagation and provides an additional fitness benefit for the host to protect against pathogens, whilst in onchocercid nematodes, apart from the mutual metabolic dependence, this bacterium is involved in moulting, embryogenesis, growth and survival of the host.

**Methods:**

This review details the molecular data of *Wolbachia* and its effect on host biology, immunity, ecology and evolution, reproduction, endosymbiont-based treatment and control strategies exploited for filariasis. Relevant peer-reviewed scientic papers available in various authenticated scientific data bases were considered while writing the review.

**Conclusions:**

The information presented provides an overview on *Wolbachia* biology and its use in the control and/or treatment of vectors, onchocercid nematodes and viral diseases of medical and veterinary importance. This offers the development of new approaches for the control of a variety of vector-borne diseases.

**Graphic Abstract:**

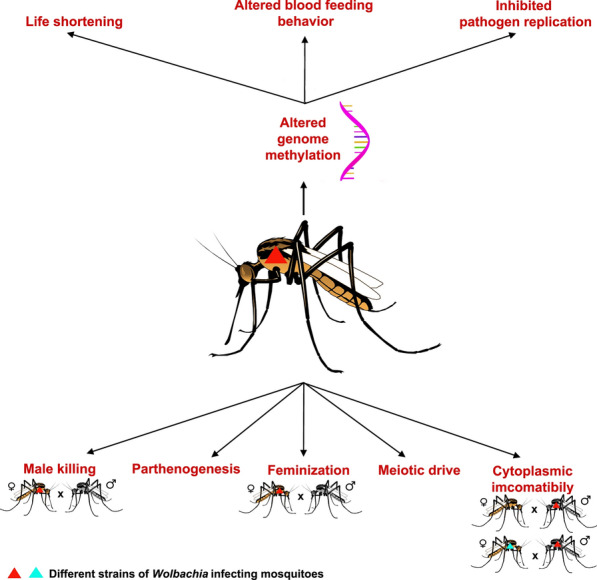

## Background

Endosymbiosis is an intimate form of symbiotic association in which one organism dwells within the body of another, forming a spectrum of relationships from parasitism to obligatory mutualism [1]. Many obligate mutual symbiotic associations are based on metabolic complementation and strengthen or increment the biochemical versatility and pathways of one or both hosts [2, 3]. *Wolbachia* is a striking example of this mechanism in both onchocercid nematodes and arthropod vectors [4]. Though many endosymbionts have been observed in arthropod and nematode hosts, *Wolbachia* is the one which is most widely distributed and explored [4]. Like the mitochondria organelle, this obligate intracellular gram-negative bacterium is also transmitted through the host germ line to the next generation [5]. After the initial discovery in the reproductive organs of *Culex pipiens* mosquito by M. Hertig and S. B. Wolbach in 1924 [6], the description of this bacterium took another 12 more years [7]. In the late 1960s and early 1970s, ultrastructural studies on filarial nematodes revealed the presence of unusual intracellular bodies in the oocyte hypodermis of these worms, which were interpreted as bacteria [8–10] and later identified as *Wolbachia* by Sironi and colleagues [11]. Currently, this endosymbiont has been reported in around 50% of terrestrial arthropod species (i.e. insects, mites, crustaceans, spiders, scorpions, collembolans) and in several species of onchocercid nematodes [12, 13]. Similarly, this bacterium has also been identified in non-filarial plant nematodes, *Radopholus similis* [14] and *Pratylenchus penetrans* of the order Tylenchida [15]. In onchocercid nematodes, *Wolbachia* has a mutual association in which it is involved in embryogenesis, moulting, growth and survival [16] of the filariae, and it has been hypothesised that the worm provides essential aminoacids for bacterial growth [17]. However, in arthropods, the “parasitic side” of *Wolbachia* prevails, in that this bacterium manipulates the host reproduction to increase its own fitness and spread into the host population [18, 19]. Reproductive manipulations exerted by *Wolbachia* on its hosts have extensively been investigated and include male killing, feminization, parthenogenesis and cytoplasmic incompatibility (CI) [4]. The effects of *Wolbachia* presence on its hosts (i.e. host biology, physiology, immunity, ecology, evolution and reproduction) have been exploited for the development of promising endosymbiont-based strategies for the treatment of filariasis and for the control of important vector-borne diseases of medical and veterinary relevance [4]. This review details *Wolbachia*'s evolution, molecular identification, interaction with arthropods and nematodes and the development of endosymbiont-based treatment and control strategies.

### Evolutionary history of *Wolbachia*

*Wolbachia* evolved and adapted to its intracellular lifestyle in the context of an evolutionary change that included other obligatory intracellular organisms (such as the ancestors of *Rickettsia, Ehrlichia, Anaplasma* and *Midichloria*) and extended over a hundred million years, starting from ancient alphaproteobacteria [20–22]. *Wolbachia* has a small genome (0.8–1.7 Mbp) with large segments of mobile and repetitive DNA, which is uncommon in vertically transmitted (generally from mother to offspring) organisms [4, 23]. Despite the erosive genomic processes due to host restriction and acquisition maintenance, these repetitive host DNA sequences are supposed to play a major role in the evolution of *Wolbachia* [24]. Balance among vertical transmission, host switching, recombination insertion sequences and bacteriophage sequences helps in the adaptation and global distribution of *Wolbachia* [4, 22]. Based on their main genetic evolution in a large variety of hosts, *Wolbachia* have been classified in 17 supergroups, designated by the letters A to S [23, 25, 26]. Exceptions are represented by supergroup G, which was lately been withdrawn because of the high probability of being the result of a genetic recombination event [27, 28], and supergroup R from cave spiders [29], which showed a strong association with *Wolbachia* strains of supergroup A, based on genetic distance measures and phylogenetic analyses [30]. Overall, *Wolbachia* of arthropods is categorised in supergroups A, B, E, H, I, K, that of nematodes in C, D, J [31] and supergroup L only in plant-parasitic nematodes [15]. Supergroup F is an exception which is common in many arthropod species such as termites, spiders, mites [32], bugs (i.e. *Cimex lectularius* and *Montina* sp.) [33] and in human filariae (i.e. *Mansonella*) [34–36]*,* filariae of black bear, (*Cercopithifilaria japonica*) [12] and that of geckoes (*Madathamugadia hiepei*) [37]. Currently, a complete genome of *Wolbachia* from the supergroup F is available from the *w*Cle strain of *C*. *lectularius* [2] and *w*Mhie strain of *M*. *hiepei* [23].

In particular, supergroups A and B are the most represented among arthropods and it is estimated that the common ancestor of both would have diverged approximately 58–67 million years ago. Though estimation of the origin of *Wolbachia* is a controversial topic and a suitable outgroup for phylogenetic analysis of *Wolbachia* is unavailable, it has been suggested that arthropod and onchocercid nematode supergroups diverged around 100 million years ago (i.e. 500 million years after their host) [38, 39]. It is important to note that these estimations were based on small samplings. The presence of *Wolbachia* in phylogenetically distant hosts such as nematodes and arthropods suggests that these endosymbionts experienced some type of horizontal transmission during their ancient evolution. For example, a horizontal transfer of *Wolbachia* could have occurred from one host phylum to the other; alternatively, one of the two phyla could have acquired *Wolbachia* from a third party [39]. Incongruence in phylogenies of *Wolbachia* and their arthropod hosts (e.g. the unnatural occurrence of identical *Wolbachia* strains in distantly related species) can also be explained by the horizontal transmission of these endosymbionts [38, 40, 41]. In addition, ecological events occurring in the transmission and global distribution of this bacterium in arthropods include the relationship between and amongst hosts, such as in the case of parasitism, phoresis [42], predation and cannibalism [43], blood contact after injury [44], presence in parasitoids [45] or just sharing of common food substrates [46].

Unlike arthropods, phylogenetic congruence of nematodes with *Wolbachia* indicates an obligatory dependent relationship with the organism, followed by host-parasite co-evolution and vertical transmission via infected females [34, 39]. Though the association between *Wolbachia* and nematodes has been hypothesised to have been acquired as a single event [34, 39], recent genome analyses suggest multiple events of acquisition of *Wolbachia* with local coevolutionary patterns in different major lineages and wider presence of transposable elements in supergroup D (i.e. in *Wolbachia* from the *Onchocerca* genus) [13, 23]. Moreover, different patterns of symbiosis among various filarial nematodes may be due to multiple acquisitions of the bacteria and/or selective pressures imposed on it [23]. The strongest coevolution pattern has been observed in *Onchocerca* spp. especially in *Onchocerca lupi*, *Onchocerca gutturosa*, *Onchocerca lienalis*, *Onchocerca volvulus* and *Onchocerca ochengi*, strongly supported by global fit analyses [13]. Furthermore, the detection of *Wolbachia*-like gene transcript in *Onchocerca fluxosa*, which in turn is the only known *Onchocerca* species devoid of *Wolbachia*, suggests the ancestral presence of this symbiont in this nematode [47, 48]. Accordingly, the absence of *Wolbachia* in anuran onchocercid nematodes indicates that they would have diversified before the first bacterial invasion in onchocercid lineage (i.e. around 110 million years ago) [25, 39, 49]. However, the presence of supergroup F in both insects and filariae of humans, black bear and geckoes makes understanding of the whole picture even more complicated.

### Molecular detection and identification of *Wolbachia*

Though the genus *Wolbachia* has a relatively small genome (i.e. 0.8–1.7 Mbp) it encompasses large phylogenetic variations [23, 39]. Amongst target genes, 16S rRNA showed a nucleotide divergence from 0.2% to 2.6% [50] but provided limited information for inferring phylogenetic relationships [50]. Hence, the genetic characterisation using 16S rRNA is complemented by a set of housekeeping genes (e.g. *FtsZ*, *groEL*, *gltA* and *coxA*) mainly for the phylogenetic analysis [31] (Table 1). *Wolbachia* surface protein (*wsp*) gene, ten times more variable than 16S rRNA and *FtsZ*, is employed to identify different groups and strains of *Wolbachia* [51, 52] but not for large-scale phylogenetic analysis since it is affected by recombination amongst supergroups [28]. Moreover, *groE* is also used for strain differentiation because of the faster evolution rate of non-coding regions that separate the coding heat shock protein (HSP) genes (i.e. *groES* and *groEL*) [53]. Therefore, PCR coupled sequencing of a combination of genes should be employed to assess the group relationships in *Wolbachia* [54]. It has been estimated that multiple infections can be detected by techniques such as quantitative PCR with highly specific primers [55, 56], cloning and sequencing [57], and southern hybridization [58]. Similarly, loop-mediated isothermal amplification (LAMP) is used in resource-limited laboratories for the simultaneous detection of more than one strain of *Wolbachia* [59]. A metagenomics-based approach can be employed to provide whole-genome sequence information for all associated endosymbionts of a nematode or an arthropod vector [60–63].

**Table 1:** Molecular approaches for the detection of *Wolbachia* in vectors and onchocercid nematodes

Primer name	Gene targeted	Type of PCR	Product size	References
99F, 99R	16S rRNA	cPCR	895	[50, 164]
Wspecf, Wspecr	16S rRNA	cPCR	438	[165]
16SWolbf, 16SWolbr	16S rRNA	cPCR	1014	[34]
INTF1, INTR	16S rRNA	cPCR	130	[166]
INTF2, INTR2	16S rRNA	cPCR	136	[166]
553F_W, 1334R_W	16S rRNA	cPCR	781	[54]
*WolbF, Wspecr*	16S rRNA	cPCR		[167]
63f, 1387R, 76f, 1012R	16S rRNA	Nested PCR	852	[168]
WN16S-F, WN16S-R	16S rRNA	qPCR		[169]
W-Specf, W-Specr,	16S rRNA	qPCR	438	[170]
W-Specf, W16S			102	
WSPintF, WSPintR	*wsp*	cPCR	576	[34, 114, 168]
81F, 691R	*wsp*	cPCR	610	[51, 171]
136 F, 691R	*wsp* (Group A)		556	
308 F, 691R	*wsp* (Subgroup Mel)		405	
328 F, 691R	*wsp* (Subgroup AlbA)		379	
173F, 691R	*wsp* (Subgroup Mel and AlbA)		541	
181F, 691R	*wsp* (Subgroup *w*Pap)		506	
165F, 691R	*wsp* (Subgroup *w*Aus)		506	
81F, 531R	*wsp* (Subgroup *w*Pap and *w*Aus)		460	
81 F, 522R	*wsp* (Group B)		442	
183F, 691R	*wsp* (Subgroup Pip)		501	
*wsp*F, *wsp*R, *gr*F, *gr*R	*wsp*	qPCR		[172]
wspTMF, wspTMR	*wsp*	qPCR		[59]
WSP.F3, WSP.B3, WSP.FIP, WSP.BIP	*wsp*	LAMP assay		[173]
FIP_wMel/wPop	*wsp* (*w*Mel/ *w*Pop)	LAMP assay		[59]
BIP_wMel/wPop				
F3_wMel/wPop				
B3_wMel/wPop				
LpF_wMel/wPop				
LpB_wMel/wPop				
ftsZ_F1, ftsZ_R1	*FtsZ*	cPCR	524	[40]
FtsZUniF, FtsZUniR	*FtsZ*	cPCR		[174]
ftsZfl, ftsZrl	*FtsZ*	cPCR	1043–1055	[38]
Wol1F, Wol1R, Wol7F, Wol7R	*FtsZ*	Nested PCR	147	[175]
MLST primers	16 s rRNA, *gatB, FtsZ, hcpA, fbpA* *coxA. wsp*	MLST		[40]
ftsZ 291, ftsZ 477	*FtsZ*	qPCR		[176]
WSP 420, WSP 583	*wsp*			
Bm-wFtsZ-F, BmwFtsZ-R	*FtsZ*	qPCR		[96]
groEL-F, groEL-R	*groEL*	cPCR		[32]
WgroF1, WgroRev1	*groEL*	cPCR	873	[35]
WgltAF1, WgltARev1	Citrate synthase (*gltA*)	cPCR	627	[35]
FbpA_F1, FbpA_R1	*FbpA*	cPCR	509	[40]
hcpA_F1, hcpA_R1	*HcpA*	cPCR	516	[40]
coxA_F1, coxA_R1	*coxA*	cPCR	487	[40]
COIintF, COIintR	*COI*	cPCR	689	[34]
Wseq01F, Wseq02R	*gatB*	cPCR	471	[31]

To date, eight complete genomes (i.e. *w*Bm of *Brugia malayi*, *w*Bp of *Brugia pahangi, w*Oo of *Onchocerca ochengi*, *w*Ov of *Onchocerca volvulus*, *w*Dimm*, Dirofilaria immitis, w*Ctub of *Cruorifilaria tuberocauda, w*Dcau of *Dipetalonema caudispina, w*Lsig of *Litomosoides sigmodontis*) and three draft genomes of *Wolbachia* from filarial nematodes have been published (i.e. *w*Lbra of *Litomosoides brasiliensis*, *w*Wb of *Wuchereria bancrofti* and *w*Mhie of *Madathamugadia hiepei*) [17, 23, 64–67]. Of the 36 complete genomes and 55 draft genomes of *Wolbachia* available, 84% belong to supergroups A and B [23]. Advanced genome analyses on *Wolbachia* suggest that supergroups A and B were originated by genetic isolation events rather than convergent evolution [68]. As per genome analyses, the relationship between onchocercid nematodes and *Wolbachia* may represent a “genetic addiction” rather than mutualism [1]. Compared to arthropods, the filarial nematode genome has smaller size (i.e. 863,427 bp for *w*Dcau versus 1,267,782 bp for *w*Mel from *Drosophila melanogaster* or 1,801,626 bp for *w*Fol from *Folsomia candida*), presence of fewer transposable elements as insertion sequence elements (ISs) and group II intron-associated genes, prophage-related genes and repeat-motif proteins as ankyrin domains [23]. Data analyses on intragenomic recombinations, transposable elements, chromosome rearrangements, mutational bias and gene loss or gain on different supergroups revealed that supergroup C strains have a very low number of genomic rearrangements, paucity of insertion sequence elements and strong GC asymmetric distribution, which is considered to be due to the long-term obligate symbiotic relationship with their host [69]. Further addition of the genome of new *Wolbachia* strains from different filarial nematodes will help to do detailed analyses and to have a clear picture on the divergent symbiotic mechanisms and the evolutionary pattern of this bacterium.

### *Wolbachia* in onchocercid nematode vectors

First reported in *Cx. pipiens* [7], *Wolbachia* is a widespread endosymbiont among arthropods with an estimated prevalence ranging from 20 to around 75%, according to different studies [38, 70]. Indeed, arthropods have been found to be infected with single (e.g. *C. lectularius*) or multiple *Wolbachia* variants (e.g. *Drosophila simulans*, *Cx. pipiens*) in the same species or even in the same insect individuals (i.e. superinfection) [71–74] (Table 2). Unlike onchocercid nematodes, arthropod-*Wolbachia* association is more parasitic, in that the bacteria obtain fitness advantage by the reproductive manipulations of the host [16, 75–77]. Apart from these, the bacterium is involved in iron homeostasis of the host and confers immunity to viral/onchocercid nematode infections, thereby reducing the vector capacity of their hosts [78–80]. This has been demonstrated in *Aedes aegypti* infected by *Wolbachia* popcorn strain (*w*MelPop) wherein the symbiosis conferred protection to the mosquitoes against onchocercid nematodes and *Plasmodium gallinaceum* parasites [79, 81]. The wide host range, tissue distribution and ability to perpetuate in insect populations render *Wolbachia* very attractive as a tool to reduce the vector potentiality of their host [82, 83] also thanks to its capability to determine reproductive manipulations (e.g. CI, parthenogenesis, male killing, feminization and meiotic drive) [84, 85]. By these phenotypic alterations, the symbiont gives more reproductive advantage to infected over uninfected individuals or genders [63]; in general, *Wolbachia* is more beneficial for the individuals of the female sex, by virtue of the matriline vertical transmission [63]. In males, *Wolbachia* affects genes involved in sex differentiation and development by altering the DNA methylation whereas in females it might interfere with steroid hormone signalling [86]. In addition to this, *Wolbachia w*MelPop infection in *Ae. aegypti* mosquitoes produces hypomethylation/demethylation of genomic DNA, affecting 699 genes involved in apoptosis, defence response, phagocytosis, circadian rhythm, life span and locomotion behaviour [87–89]. The above changes may be capitalized as a mean for controlling vector-borne diseases [90] through life-shortening and increased locomotor activity and metabolism of mosquito vectors [90]. Overall, the above factors may indeed alter the mosquito-pathogen relationship reducing the vectorial capacity of mosquitoes. In addition, the ability of *Wolbachia* to inhibit the replication of viral RNA can be used to suppress virus infection by transinfecting mosquitoes with proper *Wolbachia* strains [78, 91]. Therefore, though the exact mechanism of such inhibition is not known, *Wolbachia* can be used as a biocontrol agent to effectively control viral pathogens such as dengue, yellow fever and chikungunya viruses and other vector-borne pathogens such as filarial nematodes and the malaria parasite, *P. gallinaceum* [92–94].

**Table 2:** Main supergroups and subgroups of *Wolbachia* detected in vectors

Arthropod	Vector	Supergroup detected	Subgroup	Gene targeted	Reference
*Aedes*	Yellow fever virus, dengue (D1, D2, D3, D4) viruses, chikungunya virus, zika virus (ZIKV), Rift Valley fever virus, *Wuchereria bancrofti*, *Brugia malayi*				
* Ae. albopictus*		A, B	AlbA, Pip	*wsp*	[83, 169, 177]
* Ae. albotaeniatus*		A	Uni, Albo	*wsp*	[83, 178]
* Ae. aegypti*		A, B	-	*wsp*, 16S rRNA	[167]
* Ae. pseudoalbopictus*			AlbA, Pip, Pseu	*wsp*	[83, 178]
* Ae*. (*Stegomyia*) spp.		A, B	AlbA, Pip	*wsp*	[83]
* Ae. niveus* subgroup A		A	Mel, Niv	*wsp*	[83, 178]
* Ae. novoniveus*		A	Riv, Uni, Nov	*wsp*	[83, 178]
* Ae. craggi*		B	CauB, Crag	*wsp*	[83, 178]
* Ae. perplexus*		B	CauB, Perp	*wsp*	[83, 178]
* Ae. pseudalbopictus*		A, B	AlbA, Pip	*wsp*	[83]
* Ae. cinereus*		B		*wsp*	[177]
* Ae. cantans*		B	_	*wsp*	[177]
* Ae. cinereus*		C	Di	16S rRNA	[179, 180]
* Ae. detritus*		C	Di	*wsp*	[181]
* Ae. geniculatus*		C	Di	*wsp*	[181]
* Ae. punctor*		B, C	Pip, Di	*wsp*	[181]
* Ae. fluviatilis*		B	Flu	16S rRNA	[182]
* Ae. cooki* * Ae. polynesiensis* * Ae. riversi*		-	-	16S rRNA	[179, 180]
* Ae. bromeliae*		A	-	*wsp*	[183]
* Ae. metallicus*		A	-	*wsp*	[183]
*Armigeres*	Japanese encephalitis virus, *Wuchereria bancrofti, Dirofilaria immitis, Brugia pahangi*			*wsp*	
* Ar. kesseli*		B	Pip	*wsp*	[83]
* Ar. subalbatus*		A	Riv, AlbA	*wsp*	[83, 171]
* Ar. flavus*		A	AlbA	*wsp*	[83]
*Anopheles*	*Plasmodium falciparum, P. vivax* *P. malariae, P. ovale, P.knowlesi*, *Wuchereria bancrofti*, *Brugia malayi*				
* An. funestus*		A, B	Anfu	*coxA, fbpA* and *FtsZ*	[147, 184]
* An. gambiae*		A, B	Anga-BF, Anga-Mali	16S rRNA	[143, 185, 186]
* An. coluzzii*		A, B	Anga-BF, Anga-Mali	16S rRNA	[143, 185, 186]
* An. arabiensis*		A, B	Anga	16S rRNA	[143]
* An. carnevalei*		A, B	–	*coxA, fbpA* and *FtsZ*	[184]
* An. coustani*		B	–	*coxA, fbpA* and *FtsZ*	[184]
* An. hancocki*		B	–	*coxA, fbpA* and *FtsZ*	[184]
* An. implexus*		B	–	*coxA, fbpA* and *FtsZ*	[184]
* An. jebudensis*		B	–	*coxA, fbpA* and *FtsZ*	[184]
* An. marshallii*		B	–	*coxA, fbpA* and *FtsZ*	[184]
* An. moucheti*		B	–	*coxA, fbpA* and *FtsZ*	[184]
* An. nigeriensis*		B	–	*coxA, fbpA* and *FtsZ*	[184]
* An. nili*		B	–	*coxA*, *fbpA* and *FtsZ*	[184]
* An. paludis*		B	–	*coxA*, *fbpA* and *FtsZ*	[184]
* An. vinckei*		A, B	–	*coxA*, *fbpA* and *FtsZ*	[184]
* An. minimus*		F, D	–	16S rRNA	[170]
* An. baimaii*		D, F	–	16S rRNA	[170]
* An. maculatus*		B, F	–	16S rRNA	[170]
* An. pseudowillmori*		B	–	16S rRNA	[170]
* An. sawadwongporni*		B	–	16S rRNA	[170]
* An. minimus*		D	–	16S rRNA	[170]
* An. dirus*		B	–	16S rRNA	[170]
*Culex*	West Nile virus, equine encephalitis virus, Japanese encephalitis virus, Saint Louis encephalitis virus, Rift Valley fever virus, *Wuchereria bancrofti*, *Brugia malayi*				
* Cx p. pipiens*		B	Pip	16S rRNA	[51]
* Cx. fuscocephala*		B	Pip, Fus	*wsp*	[83, 178]
* Cx. gelidus*		B	Con, Gel	*wsp*	[83, 178]
* Cx. quinquefasciatus*		A, B	Pip	*wsp*	[83, 169, 171]
* Cx. sitiens*		B	Pip, Con, Sit	*wsp*	[83, 178]
* Cx. vishnui*		A, B	Riv, Con	*wsp*	[83]
* Cx. brevipalpis*		A	Mors, Riv, Uni, Bre	*wsp*	[83, 178]
* Cx. (Eumelanomyia)* spp*.*		A	Eum	*wsp*	[178]
* Cx* (*Lophoceraomyia*) spp.		A	Lop	*wsp*	[178]
* Cx. modestus*		B	Pip	*wsp*	[178]
* Cx. torrentium*		B, C	Pip*,* Di	*wsp*	[178]
*Conquillettidia*	West Nile virus. Eastern equine encephalomyelitis virus, John Cunningham virus				
* Cq. richiardii*		B	Con	*wsp*	[177, 181]
* Cq. crassipes*		A, B		*wsp*	[83]
*Hodgesia* spp.	-	A	Uni	*wsp*	[83]
*Mansonia*	Rift Valley fever virus			*wsp*	
* Mn. indiana*		A, B	Riv, Con	*wsp*	[83]
* Mn. uniformis*		A, B	Riv, Con, Pip	*wsp*	[83, 171]
* Mn. africana*		B	–	*wsp*	[183]
*Tripteroides aranoides*	Sylvan yellow fever virus	B	Pip	*wsp*	[83]
*Uranotaenia patriciae*	Not known (isolated Eastern equine encephalitis virus, cyprovirus, Nounane virus)	A, B	Mors, Uni, Pip	*wsp*	[83]
*Phlebotomus*	*Leishmania* spp., *Bartonella* spp., phlebovirus, Toscana virus				
* P. papatasi*		A	Turk 54, pap	*wsp*	[187]
* P.* (Larroussius) *perfiliewi*		B	AZ2331	*wsp*	[187]
*Paraphlebotomus*	*Leishmania major*			*wsp*	
* Pa. mongolensis*		A	Turk 07, Turk 54	*wsp*	[187, 188]
* Pa. caucasicus*		A	Turk 07, Turk 54	*wsp*	[187, 188]
*Lutzomyia*	*Leishmania* spp.				
* Lu. c. cayennensis*		B	Lev*,* Lcy	*wsp*	[189]
* Lu. dubitans*		B	Lev	*wsp*	[189]
* Lu. evansi*		B	Lev	*wsp*	[189]
* Lu. cruciata*		–	–	*wsp*, 16S rRNA	[190]
* Lu. trapidoi*		A	–	*wsp*	[191]
* Lu. vespertilionis*		A	–	*wsp*, FtsZ	[191]
*Culicoides*	Bluetongue virus, African horse sickness virus, Schmallenberg virus				
* C. pulicaris*		A, B	–	*wsp*	[192]
* C. imicola*		A, B	–	*wsp*	[192]
* C. kibunensis*		B	–	*wsp*	[192]
* C. vexans*		B	–	*wsp*	[192]
* C. obsoletus*		B	–	*wsp*	[192]
* C. narrabeenensis*		B	–	16S rRNA	[193]
*Simulium*	*Onchocerca* spp.		–		
* Simulium squamosum*		No identity with any of the supergroups	Dam	*aspC, aspS, dnaA, fbpA, ftsZ, groEL, hcpA, IDA, rpoB, rpe, TopI wsp, FtsZ*	[194, 195]
*Glossina*	*Trypanosoma* spp.				
* G. morsitans*		A	Mors	*wsp*	[51]
* G. austeni*		A	Aus	*wsp*	[51]
*Haematobia irritans irritans*	Filarial nematode, *Stephanofilaria stilesi, Staphylococcus* spp.	A	wIrr	Whole genome sequencing	[163]
*Tabanus* sp.	Parasite transportation (*Dermatobia hominis*), biological transmission (*Loa loa*), and mechanical transmission of viruses, (equine infectious anemia virus), protozoa, (*Trypanosoma evansi*, *Besnotia besnoiti*) and bacteria (*Bacillus anthracis*, *Anaplasma marginale*)	–	–	*FtsZ*	[38]
Muscidae	Viruses (polioviruses, coxackie viruses), numerous bacteria (*Campylobacter jejuni*, *Helicobacter pylori*, *Salmonella* sp., *Listeria* sp., *Yersinia pseudotuberculosis*, *Shigella*, *Vibrio*), protozoan parasites (*Giardia*, *Entameba*) and eggs of several tapeworms				
* Musca sorbens*		A	–	*wsp*	[196]
* Musca domestica*		B	–	*wsp*	[196]
Calliphoridae	Myiasis-producing agent, mechanical transmission of eggs of *Taenia* sp., *Entamoeba coli*, *Giardia lamblia*, *Mycobacterium paratuberculosis*				
* Chrysomya megacephala*		A, B	–	*wsp*	[196]
* Hemipyrellia pulchra*		A	–	*wsp*	[196]
Sarcophagidae	Myiasis-producing agent				
* Sarcophaga dux*		A	–	*wsp*	[196]
* Sarcophaga scopariiformis*		B	–	*wsp*	[196]
Fleas	*Yersinia pestis*, *Rickettsia* spp., *Bartonella* spp., *Dipylidium caninum*, *Hymenolepis diminuta*,				
* Ctenocephalides felis*		I	–	16S rRNA	[32, 35, 197]
*Tunga penetrans, Pulex irritans, P. simulans, Echidnophaga gallinacea, Ctenocephalides canis* *Xenopsylla brasiliensis* *Xenopsylla cheopis* *Xenopsylla nubica*		Not specified	–	16S rRNA	[197–202]
Bugs	*Trypanosoma cruzi, Bartonella quintana Burkholderia multivorans*				
*Cimex lectularius*		F	–	16S rRNA, *FtsZ*	[203]
*Montina* sp. (Reduvid bug)		-	–	*FtsZ*	[38]
Ticks					
*Rhipicephalus microplus*	*Babesia bovis,* *Babesia bigemina*	A	–	16S rRNA	
*Ixodes ricinus*	*Borrelia burgdorferi*, *Anaplasma phagocytophilum*, tick-borne encephalitis (TBE) virus	A			[204, 205]
*Gustavia microcephala* (Oribatid mite)	*Anoplocephala* spp. and *Mesocestoidea* spp.	E	–	16S rRNA, *Fts*Z*,* and *gltA*	[206]

### *Wolbachia* in onchocercid nematodes

**Fig. 1:**
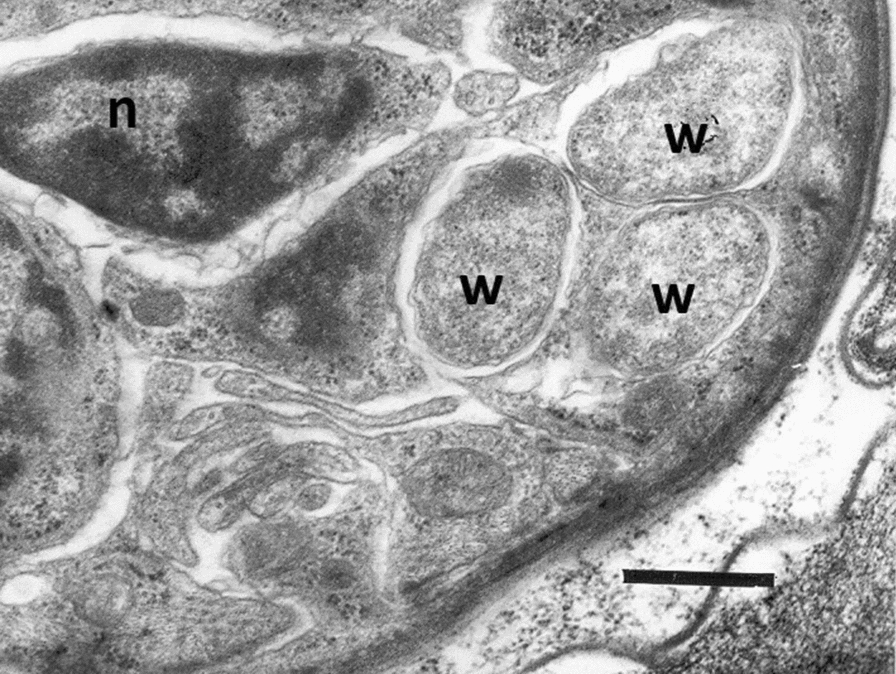
*Wolbachia* in an embryo of the nematode *Dirofilaria immitis* (transmission electron microscopy observation). W: *Wolbachia* bacteria; n: nucleus; scale bar: 0.6 µm (Photograph of Luciano Sacchi and Claudio Bandi, Modified from Bergey's Manual of Systematic Bacteriology, volume 2: The Proteobacteria) [221]

*Wolbachia* are obligatory endosymbionts required for the reproduction, development and long-term survival of onchocercid nematodes [95] and it has been hypothesized that they get from them, in return, essential aminoacids [17]. This endosymbiont is present in gradually increasing density from L1 to the adult filarioid [16, 96] of three subfamilies of Onchocercidae (i.e. Onchocercinae, Dirofilariinae and Splendidofilariinae) and in 16 of the 26 genera examined [12, 13, 49, 97] (Table 3). Indeed, most species in *Litomosoides* or *Onchocerca* genera have been found infected with *Wolbachia* but only one in the genus *Cercopithifilaria* (i.e. *Cercopithifilaria japonica*) [49, 97, 98]. However, there is a huge diversity in the localisation of these bacteria in tissues of different onchocercid nematodes and even between male and female individuals within the same species [97, 99]. In general, this bacterium is present in the female reproductive system and throughout embryonic development in the uterus of females (Fig. 1) being vertically transmitted to the progeny through the egg cytoplasm [49, 97, 100]. Other target tissues are the hypodemal lateral cords of the majority of onchocercid nematodes (e.g. *Onchocerca, Brugia, Dirofilaria*) and the intestinal cells of *Mansonella* (i.e. supergroup F) [12]. Nonetheless, the presence of *Wolbachia* may vary based on the species of onchocercid nematodes, being absent/less dense in lateral cords of *Loxodontofilaria caprini* [101] and *Onchocerca dewittei japonica* [97, 99]. Therefore, though the distribution of this endosymbiont is mostly concentrated in lateral cords and reproductive organs of the host, it exhibits different tropism to tissues during embryogenesis [12, 102]. In supergroups C and D there are similar patterns of embryonic segregation of *Wolbachia* with vertically transmitted bacteria reaching the lateral cords of the embryo by asymmetric mitotic segregation up to the ovaries [102, 103]. Though many investigations have focussed on the effects exerted by *Wolbachia* on the life performances of onchocercids, the role of this endosymbiont is still unclear [75, 102]. Five biosynthetic pathways (e.g. heme, riboflavin, FAD, glutathione and nucleotide synthesis) are present only in *Wolbachia* but not in any other rickettsiales or in onchocercid nematode hosts [17] as some involved genes (e.g. heme-biosynthesis genes) are absent in the onchocercid genome [64, 104]. In addition, the whole genome sequencing of *Wolbachia* from *Brugia malayi* (*w*bm) gave some clues regarding the role played by this bacterium in the filarial life cycle [17]. For example, the presence of heme metabolism and/or riboflavin genes in the *w*bm genome suggested a role of this bacterium in iron metabolism of the onchocercid nematodes [17, 104] though the transport, degradation and regulation of heme within filarial parasites remain a mystery for the scientific community. Therefore, the inhibition of nematode moulting following an antibiotic therapy targeting *Wolbachia* has been linked to the lack of production of ecdisone-like hormones because of the absence of heme, involved in the biochemical paths above [17, 105]. Similarly, heme inhibitors interfered with the vitality of onchocercid nematodes [106]. Though heme and nucleotide biosynthetic pathways are more conserved in all *Wolbachia* genomes, pathways like vitamin B are more variable in onchocercid nematodes (e.g. the hypothesis of *Wolbachia* providing vitamin B7 is clear in some insects such as bedbugs or grasshoppers but not demonstrated in filarial nematodes) [23]. In addition, the absence of *Wolbachia* and of any other biosynthesis pathways in the genome of *Loa loa* [107, 108] might suggest the presence of other alternative pathways for the essential nutritional requirement for this onchocercid species, a still open question that needs to be addressed by the scientific community along with the screening of new drug targets for filarial control.

**Table 3:** Onchocercid nematodes, their hosts and location, vectors and the main supergroup of *Wolbachia* detected

Onchocercids	Host	Location	Vector	*Wolbachia* supergroup	References
Onchocercinae					[102]
* Acanthocheilonema dracunculoides*	Canids	Coelomic cavity and subcutaneous tissue	*Hippobosca* *Heterodoxus*	NA	[102]
* Acanthocheilonema reconditum*	Canids	Subcutaneous tissue	*Ctenocephalides* *Pulex* *Heterodoxus*	Absent	[102]
* Acanthocheilonema viteae*	Rodents	Subcutaneous tissue	Ornithodoros	Absent	[102]
* Acanthocheilonema odendhali*	Northern fur seal	–	–	Absent	[13]
* Brugia malayi*	Humans	Lymphatic system, lymph nodes, testes	*Mansonia* *Anopheles* *Aedes*	D	[102]
* Brugia pahangi*	Dogs, felids	Lymphatic system, lymph nodes, testes	*Anopheles* *Aedes*	D	[102]
* Brugia timori*	Humans	Lymphatic system, lymph nodes, testes	*Anopheles* *Aedes*	D	[102]
* Cercopithifilaria grassii*	Dogs	Subcutaneous tissue	*Rhipicephalus* spp.	NA	[102]
* Cercopithifilaria japonica*	Ursidae (Black bear)	Oesophageal and tracheal connective tissue	–	F	[12]
* Cercopithifilaria crassa*	Sika deer	Dermis	Ixodid ticks	Absent	[12]
* Cercopithifilaria longa*	Sika deer	Subcutaneous connective tissues between muscles and skin of limbs and trunk	Ixodid ticks	Absent	[12]
* Cercopithifilaria minuta*	Japanese serow	Skin	Ixodid ticks	Absent	[12]
* Cercopithifilaria multicauda*	Japanese serow	Skin	Ixodid ticks	Absent	[12]
* Cercopithifilaria roussilhoni*	Brush–tailed porcupine	Skin	Ixodid ticks	Absent	[12]
* Cercopithifilaria shohoi*	Japanese serow	Skin	Ixodid ticks	Absent	[12]
* Cercopithifilaria tumidicervicata*	Japanese serow	Skin	Ixodid ticks	Absent	[12]
* Litomosa westi*	Rodents	Abdominal and pleural cavities	*Ornithonyssus spp.*	D	[49]
* Litomosoides sigmodontis*	Rodents	Coelomic cavity	*Ornithonyssus*	D	[102]
* Litomosoides taylori*	Water nectomys	Abdominal cavity	Suspected to be mites or bat flies	D	[12]
* Litomosoides braziliensis*	Bats	Abdominal cavity	Suspected to be mites or bat flies	D	[13, 49, 207]
* Litomosoides solarii*	Bats	Abdominal cavity	Suspected to be mites or bat flies	D	[13, 207]
* Litomosoides hamletti*	Bats	Abdominal cavity	Suspected to be mites or bat flies	D	[49, 207]
* Litomosoides galizai*	Murids	Coelomic cavity	*Bdellonyssus bacoti*	D	[49]
* Litomosa chiropterorum*				Absent	[12]
* Litomosa yutajensis*				Absent	[12]
* Litomosoides chagasfilhoi*	Mongolian gerbils	Abdominal cavity	*Ornithonyssus bacoti*	D	[208]
* Loxodontofilaria caprini*	Serows	Subcutaneous tissue mainly of limbs	*Simulium japonicum* *T. japonensis* (suspected vector)	C	[12, 209]
* Montanema martini*	Typical striped grass mouse	Skin	Ixodid ticks	Absent	[12]
* Mansonella ozzardi*	Humans	Coelomic cavity	*Culicoides* *Simulium*	F	[49, 210]
* Mansonella perstans*	Humans and monkeys	Coelomic cavity	*Culicoides*	F	[102, 210]
* Mansonella streptocerca*	Humans and monkeys	Intradermal	*Culicoides grahamii*	NA	[102, 210]
* Mansonella perforata*	Sika deer	Dermis	*Culicoides* spp.	F	[12, 210]
* M. (T.) atelensis amazonae*	Primates, Cebidae	Subscapular region	*Culicoides* spp.	F	[12, 210]
* Onchocerca cervicalis*	Equids	Nuchal ligament	*Culicoides*	C	[102]
* Oncho*cerca *gutturosa/ Onchocerca lienalis*	Bovids	Nuchal ligament, connective tissue, gastro splenic ligament	*Simulium arakawae Simulium daisense Simulium kyushuense* *Culicoides*	C	[12, 209]
* Onchocerca lupi*	Canids	–	Unknown	C	[102]
* Onchocerca ochengi*	Bovids	Intradermal	*Simulium*	C	[102]
* Onchocerca volvulus*	Humans	Subcutaneous tissue	*Simulium*	C	[102]
* Onchocerca armillata*	Bovids occasionally camel	Thoracic aorta	Midges *(Culicoides),* Blackflies *(Simulium*)	C	[12]
* Onchocerca borneensis* n. sp*.*	Suids	Footpads of the hind limbs		C	[12]
* Onchocerca dewittei japonica*	Suids	Nodular fibrous structures in the footpads of fore- and hind limbs	*Simulium bidentatum*	C	[12, 207, 209]
*Onchocerca caprini*	Bovids	Skin	*Simulium* sp*.*	C	[12]
* Onchocerca suzukii*	Bovids	Subcutaneous tissue of the body, mainly in the thoracic area and pelvic limbs	*Simulium japonicum* *Prosimulium sp.* (Suspected vector)	C	[12, 209]
* Onchocerca cervipedis*	Cervids	Subcutaneous tissues of the legs	*Prosimulium impostor Simulium decorum Simulium venustum*	C	[211]
* Onchocerca boehmi*	Equids	Arteries and veins of the limbs	Not known	C	[212]
* Onchocerca skrjabini*	Cervids, bovids	Subcutaneous tissues of muzzle, hocks and to a lesser extend in brisket and shoulder	*Simulium arakawae, Simulium bidentatum, Simulium oitanum* (Putative vector)	C	[12, 209]
* Onchocerca eberhardi*	Cervids	Carpal ligament	*Simulium arakawae, Simulium bidentatum, Simulium oitanum* (Putative vector)	C	[12, 209]
* Onchocerca gibsoni*	Bovines	Subcutaneous and intermuscular nodules	*Culicoides* spp*.*	C	[34]
* Onchocerca fasciata*	Camels	Subcutaneous tissue and nuchal ligament	Unknown	–	[213]
* Onchocerca jakutensis*	Red deer, humans	Tissues of the outer thigh and the caudal part of the back; eye, neck and face nodules		–	[214]
* Wuchereria bancrofti*	Humans	Lymphatic system, lymph nodes, testes	*Culex* *Anopheles* *Aedes*	D	[102]
* Dipetalonema gracile*	Capuchin monkey	Abdominal cavity	*Culicoides* spp.	J	[13, 207, 215, 216]
* Dipetalonema robini*	New world monkey	Peritoneal cavity	*Culicoides* spp.	J	[13, 207, 216]
* Dipetalonema caudispina*	New world monkey	Peritoneal cavity	*Culicoides* spp.	J	[13, 207, 216]
* Dipetalonema graciformis*	New world monkey	Peritoneal cavity	*Culicoides* spp.	J	[216, 217]
* Malayfilaria sofiani*	Tree shrews	Tissues surrounding the lymph nodes of the neck	Not known	D	[207]
* Cruorifilaria tuberocauda*	Capybara	Kidney	Not known	J	[13]
* Yatesia hydrochoerus*	Capybara	Skeletal muscle	*Amblyomma* sp?	J	[13]
*Breinlia (Breinlia) jittapalapongi*	Tanezumi rat	Peritoneal cavity	–	Absent	[13]
Dirofìlariinae					
* Dirofilaria immitis*	Canids, felids	Right ventricle pulmonary artery	*Aedes*	C	[102]
* Dirofilaria repens*	Canids, felids	Subcutaneous tissue	*Culex* *Aedes*	C	[102]
* Loa loa*	Humans	Subcutaneous tissue	*Chrysops*	Absent	[102]
* Foleyella candezei*	Rainbow agama	Subcutaneous tissue		Absent	[12]
* Pelecitus fulicaeatrae*	Black-necked grebe	Ankle region	Lice	Absent	[13]
Setariinae					
* Setaria equina*	Horses	Coelomic cavity	*Aedes*	Absent	[102]
* Setaria tundra*	Roedeer	Peritoneal cavity	*Aedes* sp., *Anopheles* sp.	Absent	[13]
* Setaria digitata*	cattle	Peritoneal cavity	*Aedes, Culex, Anopheles, Hyrcanus, Armigeres*	Absent	[12]
Splendidofilariinae					
* Cardiofilaria pavlovskyi*	Eurasian golden oriole	Body and pericardial cavity	–	NA	[13]
* Madathamugadia hiepei*	Turner's thick-toed gecko	–	*Phlebotomus* sp*.*?	F	[13]
* Aproctella* sp. 1	Rufous-bellied Thrush Green-winged saltator	–	–	Absent	[12]
Icosiellinae					
* Icosiella neglecta*	Marsh frog, Edible frog	Muscle, subcutaneous tissue	–	Absent	[13]
Oswaldofilariinae					
* Oswaldofilaria petersi*	Crocodilurus	Mesentery, intestine and thigh muscles	–	Absent	[13]
* Piratuba scaffi*	Lizard jungle runner	Skin		Absent	[12]
Waltonellinae					
* Ochoterenella phyllomedusa*	Toads	–	*Culicine* mosquitoes	Absent	[13]
* Ochoterenella royi*	Cane toad	–	–	Absent	[12]
* Ochoterenella* sp. 1	Giant leaf frog	–	–	Absent	[12]

### *Wolbachia* as a modulator of host inflammation and immunity

**Fig. 2:**
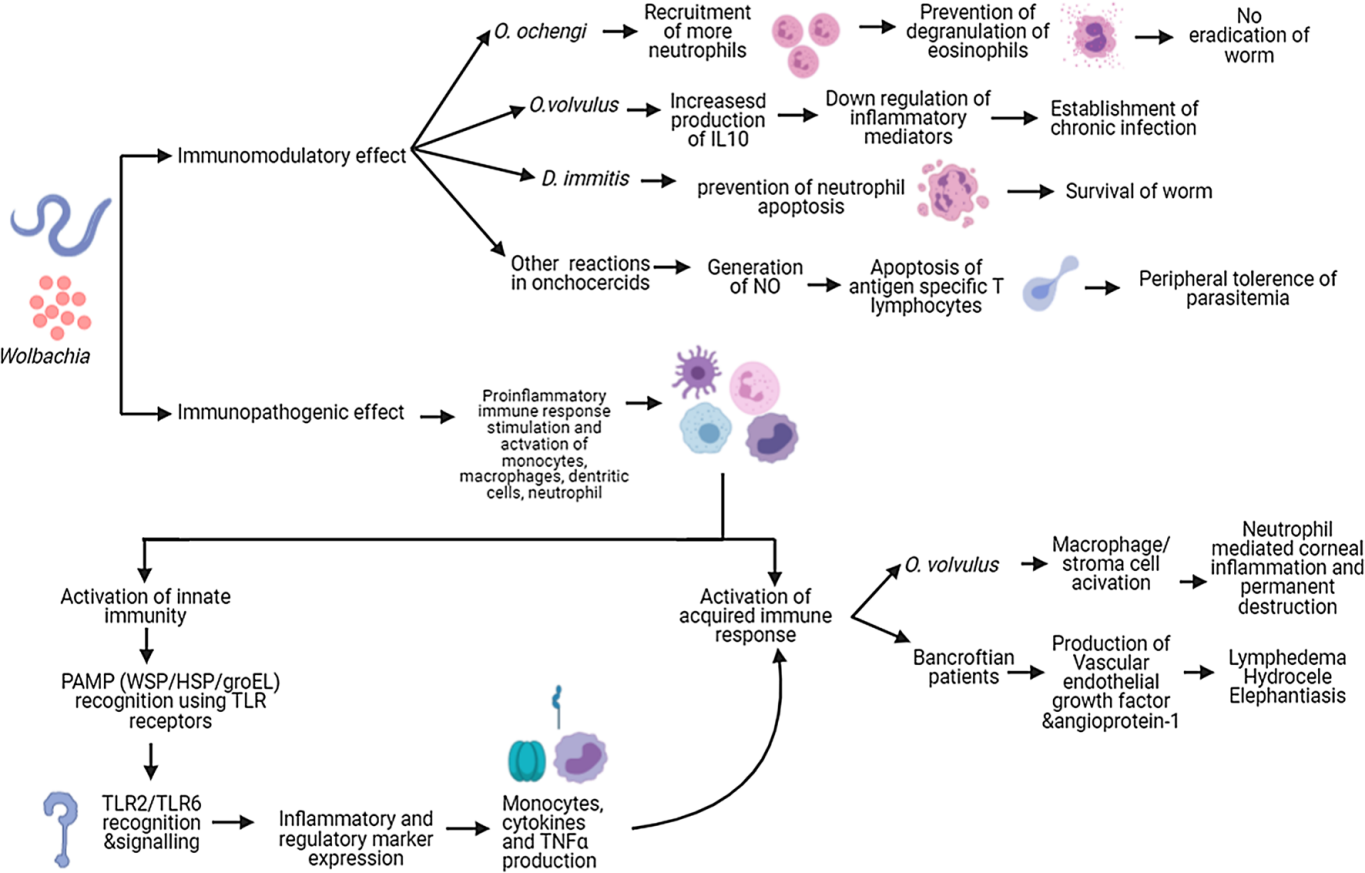
Role of *Wolbachia* in onchocecid nematode infections. *Wolbachia* induced changes in the host immune system such as immunomodulation for the survival of nematodes and various steps in the development of the immunopathology in filarial diseases are summarized

Filariasis has an extremely complex immunopathology with adult parasites surviving in immune competent patients for many years [49]. Many filarial nematodes harbour *Wolbachia* at all stages of their life cycle [49] except a few species such as *L. loa, Acanthocheilonema viteae, O. flexuosa* and *Setaria equina* [106, 109]. One of the main concerns in the treatment of filarial worms is related to the host inflammatory response provoked by the death of adult or larval worms within the parasitized tissues [77]. Under the above circumstances, the use of doxycycline targeting *Wolbachia* causes a *soft killing* of onchocercid nematodes with a slow death of adult parasites (over 12–24 months), preceded by a block of embryogenesis and larval development with clearing of microfilariae from blood or skin, thus preventing the pathology [110–112]. However, *Wolbachia* also plays a major role in the pathogenesis of both acute and chronic filariasis, which may cause severe systemic adverse reactions to chemotherapy as well as ocular inflammation by activating pro-inflammatory and immunomodulatory mechanisms in the host in cases of *O. volvulus* infection [113] (Fig. 2). Hence, *Wolbachia* has multiple roles in filariasis (i.e. activation of proinflammatory pathogenesis, immunomodulation of the host and survival of the parasite) starting from the entry of the parasite to the establishment of the infection [102].

*Wolbachia* stimulates a proinflammatory immune response to onchocercid nematodes by interacting with the host monocytes, macrophages, dendritic cells and neutrophils [77]. Indeed, *Wolbachia* may elicit a host immune response by activating the innate and adaptive responses in human and murine models of filariasis infection [102]. Generally, in bacterial infections, the host innate immune system recognises pathogen-associated molecular patterns (PAMP) associated with bacteria which are presented by pattern recognition receptors (PRP) including the Toll-like receptors (TLR) located on the surface of antigen-presenting cells [77] (Fig. 2). Though lipopolysaccharides (LPS) act as a major PAMP in most bacterial infections, the absence of genes required for LPS biosynthesis in the *Wolbachia* genomes suggests that the *Wolbachia* surface protein (WSP) is a predominant PAMP involved in the immunopathology of filarial diseases [106, 114, 115]. Recently, further evidence on the immune-modulating property of WSP showed that the *Asaia* endosymbiotic bacterium engineered for the expression of this protein induces classical macrophage activation, associated with the killing of *Leishmania* parasites [116].

In addition to WSP, other PAMPs of *Wolbachia* include HSP 60 and groEL, and all these molecules mainly elicit TLR 2 or TLR 6 recognition and signalling [77]. In particular, WSP produces inflammation and regulatory marker expression (i.e. T lymphocyte antigen 4) while HSP 60 acts on monocytes and produces cytokines and TNFα, which induce an adaptive immune response against onchocercid nematodes [102, 113, 117]. Hence, in *O. volvulus* pathology, TLR 2 activates macrophages and local stromal cells contributing to the neutrophil-mediated corneal inflammation and permanent destruction of the cornea [118, 119]. In chronically ill patients, repeated invasion of larvae and their death produce inflammatory cell influx, eventually causing permanent tissue damage by neutrophil degranulation and the production of cytotoxic products such as nitric oxide (NO), myeloperoxidase and oxygen radicals [119]. It is believed that, in bancroftian patients, *Wolbachia* with its TLR2 signalling triggers vascular endothelial growth factor A and angiopoietin-1 production, which produces the dilatation of scrotal lymph vessels [120] (Fig. 2). This is supported by the results obtained after the administration of doxycycline [121]. Absence of such immunological response by the extracts of the worm *A. viteae*, which have no *Wolbachia* endosymbiont, supports the above findings [113, 117].

In addition to the contribution in immunopathology, *Wolbachia* plays a defensive mutualistic role in filarial biology (e.g. *O. ochengi*) by triggering the recruitment of more neutrophils, which will help to prevent the degranulation of eosinophils needed for the eradication of filarial worms [122]. A similar reaction is also observed in human *D. immitis* infection wherein WSP prevent the neutrophil apoptosis [123]. Moreover, further *in vitro* studies on blood cells from patients with *O. volvulus* suggested that chronic *Wolbachia* stimulation may cause the downregulation of pro-inflammatory mediators by increasing the production of interleukin 10 (IL-10) and thus help in establishing chronic infection [124]. In addition, it is suggested that together with filarial antigens, *Wolbachia* may induce generation of NO, which aids in the peripheral tolerance through apoptosis of antigen-specific T lymphocytes [125]. This manipulation of the host immune system helps to increase the longevity of onchocercid nematodes [126, 127] (Fig. 2). Moreover, a stronger immune reaction in response to the release of L3 larvae than to the dead worm indicates the predominant role of these bacteria at the development of the early stage of the worm [77]. *Wolbachia* with its TLR 2-dependent signalling helps the filarial L3 larva establishment by surpassing chemokine (c–c motif) ligand 17 (CCL 17)-mediated immune response of the host [128].

### Exploiting *Wolbachia* for treatment

Current filariasis treatment control and Mass Drug Administration (MDA) programmes are focussed on the use of microfilaricides such as albendazole combined with either ivermectin or diethylcarbamazine [129]. Though this treatment regime helped to reduce the number of human cases of filariases, the long duration of treatment (e.g. 17 years for onchocerciasis and 5 years for lymphatic filariasis), the development of resistance to ivermectin in endemic areas as well as adverse reactions to ivermectin treatment in certain epidemiological conditions (e.g. onchocerciasis-loasis co-endemic areas) hampered the global elimination of the diseases [110, 130]. Hence, chronic debilitating pathological alterations and the economic burdens in endemic countries due to long-term treatment and control programmes highlighted the need for an alternative effective short-term potential drug target for filariasis.

Based on the unique obligatory symbiotic relationship *Wolbachia* has established with these onchocercid nematodes (e.g. embryogenesis and moulting) and the role of these bacteria in the immunopathology of filarial diseases, a major mission of the anti-*Wolbachia* (A∙WOL) consortium was to exploit the *Wolbachia-*filarial biology for controlling human infection [131]. Studies suggested that anti-*Wolbachia* therapy has both macrofilaricidal (i.e. death of adult parasites and developmental retardation) and microfilaricidal embryotoxic activity. Indeed, antibacterial agents such as doxycycline were found to be effective in clearing microfilarial stages from the blood and skin of patients, therefore preventing filarial pathology and reducing the transmission [131]. Moreover, the slow death of adult parasites over a period of 12–24 months in patients treated with doxycycline is safe to use in geographical regions were onchocerciasis and loiasis occurred in sympatry [110–112]. However, the mechanism of action of doxycycline in the treatment of filariases was not well understood until transcriptomic and proteomic analysis unveiled that the responses of *Wolbachia* to doxycycline cause impairment of bacterial metabolism [132]. Meta-analytical modelling suggests that a 4-week doxycycline course suffices to eliminate *Wolbachia* with low chances of developing drug resistance when compared to other antibiotics like penicillin or fluoroquinolones [133, 134]. However, the limited use of this drug in pregnancy, lactating mothers and children motivated the scientific community to search for other anti-*Wolbachia* drugs [127, 131]. As a result, more than 2 million compounds have been tested in insect cell lines and A∙WOL was formed with the objective to identify new anti-*Wolbachia* drugs with a short course of therapy, which could be safe in contraindicated groups [135, 136]. Mass screening of all registered antibacterials revealed four drugs as superior to doxycycline with minocycline as the most effective drug of choice [137]. Recent experimental trials claim that the use of Tylosin A, a macrofilaricide, is superior to tetracycline antibiotics (e.g. doxycycline and minocycline) and will help to reduce the duration of treatment from 3–4 to 1–2 weeks [131].

### Targeting *Wolbachia* for vector control

**Fig. 3:**
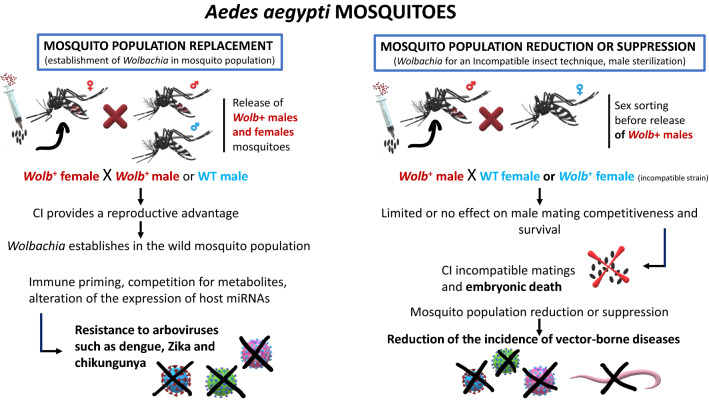
Exploiting *Wolbachia* for vector control. Left panel: mosquito population replacement approach, in which *Wolbachia*-infected female and male mosquitoes are released; through CI, this strategy allows the spread of *Wolbachia* in the natural population. The presence of *Wolbachia* provides a fitness advantages (determined by CI) and can reduce the arbovirus transmission. Right panel: mosquito population reduction or suppression strategy. This approach involves the release of *Wolbachia*-infected males into an area; when these mosquitoes mate with wild *Wolbachia*-negative females (or female mosquitoes harbouring an incompatible strain of *Wolbachia*), a strong reduction in the rate of egg hatching is observed (CI incompatible matings). Thus, repeated releases of *Wolbachia*-infected males result in reduction or suppression of mosquito populations. *CI* cytoplasmic incompatibility, *WT* wild-type mosquitoes, *Wolb +* *Wolbachia*-infected mosquitoes

Vector control methods mainly focus on the physical removal of their breeding sites in the environment or on- and off-host application of insecticides targeting immature or adult stages [138]. A combination of factors such as human population growth, globalization, rapid rise in population-dense towns, expansion of the geographical range of vectors and development of insecticide resistance affected the control of vectors and associated pathogens [139, 140]. Hence, the search for an alternative vector control approach may target either reducing the vector population or modifying the vector to make it refractory to pathogen transmission [138]. For example, the abundance of *Wolbachia* among vectors and its high rate of maternal transmission conjoined with CI have spurred the interest of researchers in new target strategies for vector control. It was discovered that *Wolbachia* can protect its natural host *Drosophila melanogaster* from pathogenic viruses, such as *Drosophila* C virus [138, 141]. Since then, many *Wolbachia* strains have been found to block the transmission of a range of medically important viruses and parasites [138, 142]. Thus, it was proposed that the use of large numbers of *Wolbachia*-infected males to sterilize local uninfected females through CI (incompatible insect technique, IIT) coupled with a pathogen-blocking *Wolbachia* strain could be effectively gradually replace the local permissive natural vectors with refractory insects [143]. Therefore, a non-profit research consortium, namely the “World Mosquito Program” (WMP), formerly known as the “Eliminate Dengue Program”, was instituted to eliminate mosquito-borne viral diseases like dengue, Zika and chikungunya [141, 144]. In this context, it has been shown that the intensity of *Wolbachia* infection is directly correlated with the strength of pathogen blocking and the tissue damage caused by the cellular load of highly replicative strains of *Wolbachia* (e.g. *w*MelPop) produces pathogen blocking in *Ae. aegypti* [145]. Other possible mechanisms are host immune priming by the preactivation of the immune response and gene regulation by the induction of Vago1 protein, which is involved in the innate immune pathways of *Culex quinquefasciatus* and *Ae. aegypti*. This mechanism could favour vectors to reduce West Nile and dengue virus replication [146, 147]. Though vectors like *Ae. aegypti* are not naturally infected with the virus inhibiting *Wolbachia*, these non-native strains of *Wolbachia* were introduced into the vectors of medical and veterinary importance by transfection [148] (Fig. 3). Other methods such as transient somatic infection, infections in cell lines, *ex* *vivo* organ culture, outcrossing and introgression can also be used for the successful introduction of *Wolbachia* in non-native vectors [147] (Table 4). Current research on control mainly focuses on limiting the susceptibility to infection rather than using this symbiont to reduce the life span of vectors [149]. Indeed, the fitness cost of *w*MelPop strain prompted the researchers to adopt another strain of *Wolbachia*, *w*Mel which does not reduce the fitness of mosquito hosts [149, 150]. Approximately 300,000 *w*Mel-infected *Ae. aegypti* mosquitoes were released in north Queensland over a period of 10 weeks [149]. The success of this strategy in north Queensland (i.e. a high infection frequency up to 80%–90%) was replicated in 12 countries, including Brazil, Indonesia, Vietnam, and four countries of the south-western Pacific region [141]. These studies have shown that the *w*Mel strain of *Wolbachia* can quickly spread to near fixation in the wild mosquito population and become stable for a long time after the initial release [138]. Apart from these, in West Africa, stable *Wolbachia* infections were recently detected in natural *Anopheles* populations and these infections appear to be negatively correlated to *Plasmodium* prevalence, which opens up the possibility of utilising these endosymbionts for the control of malaria transmission [143]. The introduction of *Wolbachia* infections in *Culicoides sonorensis* cell lines and the upregulation of immune genes in the same vectors suggested the utility of using *Wolbachia* as a bio-control agent in the transmission of *C. sonorensis* vectored pathogens of veterinary importance (e.g. African horse sickness virus, Schmallenberg virus, bluetongue virus, epizootic hemorrhagic disease virus) [151]. Like any other modern technologies, *Wolbachia*-based vector control also has some potential vulnerabilities such as (i) loss of attenuation of *Wolbachia* infection in the mosquito, (ii) emergence of virus strains that are resistant to *Wolbachia*-mediated blocking, (iii) increasing virulence and disease pathogenesis in humans, (iv) enhancement of the arbovirus infection in transfected mosquitoes and (v) development of mutations in viruses over time that render them less susceptible or resistant to *Wolbachia* [141]. Apart from these the current method of control requires continual release of large numbers of males to suppress the mosquito population and the migration of mosquitoes from the untreated surroundings will hinder the long-term effectiveness of this method. Since only modified males are released into the environment, adoption of an effective sex sorting system is required [138]. Nonetheless, none of these modifying technologies has yet been approved by the WHO’s Vector Control Advisory Group [138]. In addition, it is highly advisable to avoid adverse effects such as the enhancement of pathogen development in coinfections (Table 4) by analysing the molecular mechanisms of *Wolbachia*-pathogen interactions before doing the field trials.

**Table 4:** Progress in *Wolbachia*-based vector modifications for control and its possible outcomes

Vector	Pathogens transmitted	Stable transfections	Strains used	Protected against pathogens	Effects/favourable outcome of transfected mosquitoes	Reversal outcome effects in transfected mosquitoes	Reference
*Aedes*	Yellow fever virus Dengue (D1, D2, D3, D4) viruses Chikungunya virus Zika virus (ZIKV) Rift Valley fever virus *Wuchereria bancrofti*, *Brugia malayi*	*Aedes aegypti* *Aedes albopictus* *Aedes polynesiensis*	*w*AlbB, *w*Mel, *w*MelPopCLA	Yellow fever virus Chikungunya *Plasmodium gallinaceum* ZIKV and ZIKV/DENV coinfection transmission blockage *Brugia pahangi*	*w*AlB-mosquitoes successfully established and reduced human dengue incidence was registred *w*Mel-mosquitoes successfully established across 66 km2 and no local dengue transmission was registered	Enhance DENV D2 in *Aedes aegypti* and *Plasmodium gallinaceum* in *Aedes fluviatilis*	[144, 147, 218]
*Anopheles*	*Plasmodium falciparum, Plasmodium vivax, Plasmodium malariae, Plasmodium ovale, Plasmodium knowlesi* *Wuchereria bancrofti*, *Brugia malayi*	*Anopheles gambiae* *Anopheles stephensi*	*w*AlbB	*Plasmodium falciparum,* modestly suppress *Plasmodium berghei* oocyst levels, *Plasmodium yoelii* at some temperatures	Modest decrease in oocyst numbers and a strong reduction in salivary gland sporozoites of *Plasmodium falciparum*	*Enhance Plasmodium yoelii* at some temperatures in *Anopheles stephensi*	[147]
*Culex*	West Nile virus Equine encephalitis virus Japanese encephalitis virus Saint Louis encephalitis virus Rift Valley fever virus *Wuchereria bancrofti*, *Brugia malayi*	*Culex tarsalis*	*w*Pip	West Nile virus (WNV)	Increasing *Plasmodium relictum* transmission stages	Enhance WNV infection in *Culex tarsalis* and *Plasmodium relictum* in *Culex quinquefasciatus*	[147, 219, 220]

## Conclusions and future perspectives

Though *Wolbachia* is a relatively well-studied endosymbiont [4], there are still lacunae in the knowledge about its exact distribution, evolution, type of symbiosis and *Wolbachia*-mediated antiparasitic mechanisms. It has been hypothesised that onchocercid nematodes may depend on *Wolbachia* for their heme metabolism [17]. For example, no new biosynthesis pathways for heme metabolism are observed in *L. loa*, which lacks *Wolbachia* [17], therefore suggesting alternative pathways or the presence of other symbionts for the essential nutritional requirement of these worms. While *Wolbachia* is already well studied, many other endosymbionts have received less attention, such as some *Spiroplasma, Cardinium, Arsenophonus* and *Flavobacetrium* species [152] and have not yet been investigated in detail. So, the metagenomics approach could help to assess the associated endosymbionts in *Wolbachia* free onchocercid nematodes. In addition, the use fluorescence in situ hybridization is advisable to assess the natural infections by *Wolbachia* [153]. Since *Wolbachia* dominant proteins expressed in each life stage of onchocercid nematodes show a gradual increase from L1 to adult [16, 96], proteomic approaches (e.g. mass spectrometry, chromatography) could be useful to assess their variation in expression in each stage in the vector and the definitive host for their survival and multiplication. This may eventually lead to exploring the type of symbiosis at each stage of the parasite life cycle, also providing insights into the *Wolbachia*-mediated antiparasite mechanisms and potential new drug targets for onchocercid nematodes of medical and veterinary significance. Despite doxycycline being adopted to treat filarial diseases, potential difficulties (e.g. drug adherence, toxicity, resistance, financial cost, contraindications in pregnant women and children) limit its use in the public health MDA programmes [127]. Promising *in vitro* drug trials with new antibiotics (e.g. berberine, rapamycin, globomycin, succinyl acetone) [106, 154–156] and the effectiveness of non-antimicrobial compounds such as anti-oxidants and anti-histamines open a new window onto filarial treatment [127]. Further clinical trials using these drugs may provide an innovative strategy for anti-*Wolbachia* treatment, eventually reducing the duration of treatment.

Overall, the prevalence of *Wolbachia* differs significantly among different climatic regions and geographic locations [157]. For example, the intensity of *Wolbachia* infections in natural *Ae. albopictus* populations was low in regions with only imported dengue cases suggesting a positive correlation with the presence of *Wolbachia* in vectors and dengue infection [157]. Based on the above, it would be necessary to have a cluster-randomized design, involving either long- or short-term vector-release trials in limited locations or in more sites, respectively, to optimize the impact of this control strategy in each geographical or climatic setting [158]. Nonetheless, *Wolbachia*-based vector control strategies to control arboviral infections targeting *Aedes aegypti* are being compromised in many endemic countries because of the co-localisation of the secondary vector *Aedes albopictus* [159]. Hence, it is important to rely also on fluorescence in situ hybridisation when reporting natural *Wolbachia* infections and not only on PCR. Furthermore, use of multiple strains of *Wolbachia* for vector transfection and integration of CI-carrying phage elements into strains that are devoid of them (e.g. *w*Au) [159] could provide more fitness benefits for the transfected vector. Under the above circumstances, transfected triple-strain infection of *Wolbachia* (e.g. *w*Mel and *w*Pip and *w*Au) into a Malaysian *Ae. albopictus* line produced self-compatibility, moderate fitness cost and complete resistance to Zika and dengue infections [160]. Apart from these, more field trials using *w*AlbB strains will also help to overcome the inability of *w*MelPop strains to establish in wild mosquito populations or the *w*Mel strains to survive at high temperatures in the field [161]. This could be considered a successful strategy to reduce the incidence of dengue in an endemic area of Malaysia after the release of *w*AlbB-infected *Ae. aegypti* [161]. Similarly, use of other endosymbionts along with *Wolbachia* will help to accelerate the control of *D. immitis* through the use of genetically engineered *Asaia* bacteria for the expression of WSP from their *Wolbachia* endosymbionts [80]. However, the untoward effects of *Wolbachia* such as irreversible biological effects and reversal outcome on disease transmission [162] should be properly addressed before clinical trials. Successful introduction of *Wolbachia* infections in *Anopheles gambiae, Anopheles stephensi* and *C. sonorensis* cell lines may give a breakthrough in the control of malaria, African horse sickness, Schmallenberg, bluetongue and epizootic hemorrhagic disease. The genome sequencing of the *Wolbachia* strain, *w*Irr, of *Haematobia irritans irritans* suggests its unique features, including the horizontal acquisition of additional transcriptionally active CI loci, which may be exploited for the biocontrol and potential insecticide resistance of horn flies [163]. Despite all the challenges, studies on *Wolbachia* and their use in the control and/or treatment of vectors, onchocercid nematodes and viral diseases of medical and veterinary importance offer new approaches which undoubtedly open new avenues for the control of a variety of vector-borne diseases.

## Data Availability

All datasets supporting the conclusions of this article are included within the article.

## Abbreviations

CI: Cytoplasmic incompatibility
PAMP: Pathogen-associated molecular pattern marker
TLR: Toll-like receptors
LPS: Lipopolysaccharides
Interleukin: IL
HSP: Heat shock protein
WSP: *Wolbachia* surface protein
LAMP: Loop-mediated isothermal amplification
NO: Nitric oxide
MDA: Mass drug administration
WMP: World Mosquito Program
